# An RT–qPCR Assay for Rapid Detection of Coxsackievirus A4

**DOI:** 10.3390/v18101120

**Published:** 2026-10-09

**Authors:** Xiaofeng Qiu, Feifei Cao, Shi Cheng, Jun Li

**Affiliations:** 1 Health Inspection Center, Hangzhou Center for Disease Control and Prevention (Hangzhou Health Supervision Institution), Hangzhou 310021, China; qxf976006@163.com (X.Q.); breezefei@126.com (F.C.); chengshi92@163.com (S.C.); 2 Zhejiang Key Laboratory of Multi-Omics in Infection and Immunity, Hangzhou 310021, China

**Keywords:** coxsackievirus A4 (CV-A4), RT–qPCR, TaqMan probe, *VP1* gene, molecular diagnosis, subgenotype C2

## Abstract

Coxsackievirus A4 (CV-A4) is a major cause of hand, foot, and mouth disease and herpangina in children, and has been linked to severe complications. However, current detection methods, including commercial RT-qPCR kits, suffer from false-negative and false-positive results. This study aimed to develop a sensitive and specific TaqMan-based RT-qPCR assay for the rapid detection of CV-A4, targeting the conserved *VP1* region covering genotypes A–D and subgenotypes C1–C5, with emphasis on the predominant C2 subgenotype in China. The assay was evaluated using a panel of enteroviruses, influenza virus, adenovirus, and rhinovirus, and compared with two commercial kits using 82 CV-A4-positive and 100 negative clinical specimens. The established assay demonstrated high specificity with no cross-reactivity, a detection limit of 2.1 TCID_50_ equivalents/mL, and excellent reproducibility (*CV* ≤ 3.03%). In clinical testing, it achieved a 100% detection rate (82/82) with no false positives within 1.5 h, outperforming the two commercial kits, which showed positive rates of 91.5% and 89.0%, and false-positive rates of 0% and 1%, respectively. These findings indicate that our RT-qPCR assay offers superior sensitivity and specificity for CV-A4 detection, particularly for the C2 subgenotype, and represents a promising tool for laboratory diagnosis and epidemiological surveillance.

## 1. Introduction

Coxsackievirus A4 (CV-A4), a member of the genus *Enterovirus* within the *Picornaviridae* family, was first identified in 1948. As a group *Enterovirus alphacoxsackie*, CV-A4 possesses a single-stranded, positive-sense RNA genome of approximately 7.4 kb that encodes a single polyprotein [1,2]. CV-A4 is a common etiological agent of hand, foot, and mouth disease (HFMD) in children [3,4] and has also been associated with herpangina, tonsillitis, bronchitis, myocarditis [5,6], acute flaccid paralysis [7], and viral meningitis [8,9]. Given its broad pathogenic potential, CV-A4 poses a significant public health concern, with outbreaks frequently affecting infants and young children worldwide [10]. Notably, CV-A4 infections are not restricted to children; a 63-year-old male with viral myocarditis complicated by hepatic dysfunction and pancreatitis due to CV-A4 infection has been reported in Japan [11]. These reports underscore the importance of accurate and rapid CV-A4 detection across all age groups.

Several studies have identified CV-A4 as a causative agent of influenza-like illness (ILI), HFMD, and herpangina [6,12,13,14,15]. In a study conducted in western Cambodia between May 2010 and December 2012, CV-A4 was detected in 1.3% of 586 outpatients (median age, 5 years; range, 1–77 years) presenting with ILI [16]. More recently, epidemiological surveillance in Hangzhou during 2023 revealed an overall EV positivity rate of 3.74% (130/3480) among ILI patients, with CV-A4 ranking as the second most prevalent genotype (0.75%, 26/3480) [17]. These data highlight the ongoing clinical relevance of CV-A4 in the post-COVID-19 era.

HFMD is a common acute infectious disease caused by multiple enterovirus (EV) types. CV-A16 and EV-A71 have historically been the two major pathogens: CV-A16 typically causes mild, self-limited disease with a localized vesicular rash, whereas EV-A71 is frequently associated with severe neurological complications and has therefore been a major focus of surveillance and vaccine development in Asia [18,19]. In recent years, however, the epidemiological landscape has changed substantially, with CV-A6 emerging as a predominant pathogen in many regions. Atypical HFMD caused by CV-A6 is characterized by more widespread skin involvement, higher fever, and post-infectious sequelae such as nail shedding, and it may also affect adults [20,21]. CV-A10 can produce clinical manifestations resembling those of CV-A6, although its disease burden varies by region and season [22]. Less common EV types, including CV-A2, CV-A4, CV-A5, CV-A8, CV-B2, CV-B5, and echovirus 11, occasionally cause HFMD or HFMD-like illness but account for only a small fraction of cases and are often underrecognized because routine assays do not specifically distinguish them [23,24,25]. The relative prevalence of these EV types differs considerably across regions, and region-specific surveillance data from China, Southeast Asia, Europe, and North America have documented distinct circulation and seasonal patterns [18,19,26,27,28,29]. A comprehensive understanding of these major and minor HFMD-associated EVs is therefore essential for accurate diagnosis, outbreak investigation, and the development of type-specific molecular detection methods such as the CV-A4 assay evaluated in this study.

Currently, the primary methods for EV detection include virus isolation and culture, serological assays, and PCR-based molecular techniques. Although virus isolation and culture are considered the “gold standard” for diagnosing viral infections, these procedures are labor-intensive and time-consuming, require specialized laboratory facilities, and have limited throughput, rendering them unsuitable for rapid and efficient diagnosis. Serological methods, while faster, often lack the discriminatory power required for accurate serotyping. In contrast, real-time fluorescent reverse transcription polymerase chain reaction (RT-PCR) offers high sensitivity, specificity, efficiency, and throughput, making it a robust tool for rapid and accurate molecular diagnosis [30]. Although a SYBR Green I-based real-time RT-PCR method has been reported [31], its nonspecific binding to double-stranded DNA compromises specificity. TaqMan probe-based assays, on the other hand, provide enhanced accuracy through sequence-specific hybridization, thereby minimizing false-positive signals.

Currently available CV-A4 detection methods [3] and commercial CV-A4 kits can misidentify or miss CV-A4 owing to cross-reactivity and sequence variation, leading to false-negative or false-positive results. Rapid and accurate CV-A4 detection is essential for clinical management, outbreak surveillance, and epidemiological monitoring, even during periods of low case counts. The assay developed in this study fills a gap in type-specific molecular diagnostics that is not met by current commercial kits.

In this study, primers and probes were designed targeting the relatively conserved *VP1* region of CV-A4 using bioinformatics software to establish a robust RT-qPCR assay. The developed method demonstrated high specificity, excellent reproducibility, and high sensitivity and is expected to facilitate research on the CV-A4 epidemic in China. Given the widespread installation of real-time PCR instruments in county-level and higher hospitals and disease control institutions across China during the COVID-19 pandemic, the assay established herein possesses strong potential for clinical translation and routine application.

## 2. Materials and Methods

### 2.1. Virus Propagation and Preparation

A local CV-A4 strain (253/Hangzhou/CHN/2014), originally isolated from a throat swab specimen collected on 1 September 2014 from a 2-year-old female patient clinically diagnosed with HFMD, was used in this study. The strain was propagated in human rhabdomyosarcoma (RD) cells and subsequently stored in our laboratory. The complete genome sequence has been deposited in GenBank under accession number PP623123. Following three freeze–thaw cycles, the cell culture supernatant was harvested by centrifugation and stored at −80 °C.

The viral titer was determined in RD cells according to the poliovirus titration protocol described in the WHO Poliovirus Laboratory Manual [32], yielding a titer of 10^7.8^ TCID_50_/mL.

### 2.2. Clinical Samples

A panel of enterovirus-positive clinical samples previously identified and preserved in our laboratory was used for specificity evaluation. This panel comprised *Enterovirus alphacoxsackie* species (CV-A2, CV-A4, CV-A5, CV-A6, CV-A8, CV-A10, CV-A16, and EV-A71), *Enterovirus betacoxsackie* species (CV-A9, CV-B1, CV-B3, E6, E7, E9, E11, E18, E21, and E30), *Enterovirus coxsackiepol* species (CV-A21 and CV-A24), and *Enterovirus deconciliis* species (EV-D68) [17,33,34]. In addition, clinical specimens positive for rhinovirus, adenovirus, and influenza virus were included [17]. From 2019 to 2023, a total of 82 CV-A4-positive specimens were collected from patients presenting with ILI in Hangzhou, China. In accordance with the national influenza surveillance program (2017), ILI samples were obtained from patients with a body temperature greater than 38 °C and cough or sore throat who had not received antiviral treatment within 3 days of onset. All clinical samples were throat swabs collected from patients with HFMD or ILI. Other sample matrices (e.g., stool, serum, and cerebrospinal fluid) were not tested.

### 2.3. Total Nucleic Acid Extraction

Viral RNA was extracted from both virus isolates and clinical samples using the Tianlong™ DNA/RNA Extraction Kit 4.0 (64 T/Kit, Lot: 25102510T035) on a GeneRotex 96 Nucleic Acid Extractor (Xi’an Tianlong Technology Co., Ltd., Xi’an, China) according to the manufacturer’s instructions. The extracted nucleic acids were eluted in 60 µL of elution buffer and stored at −80 °C until further analysis.

### 2.4. Primer and Probe Design

**Figure 1:**
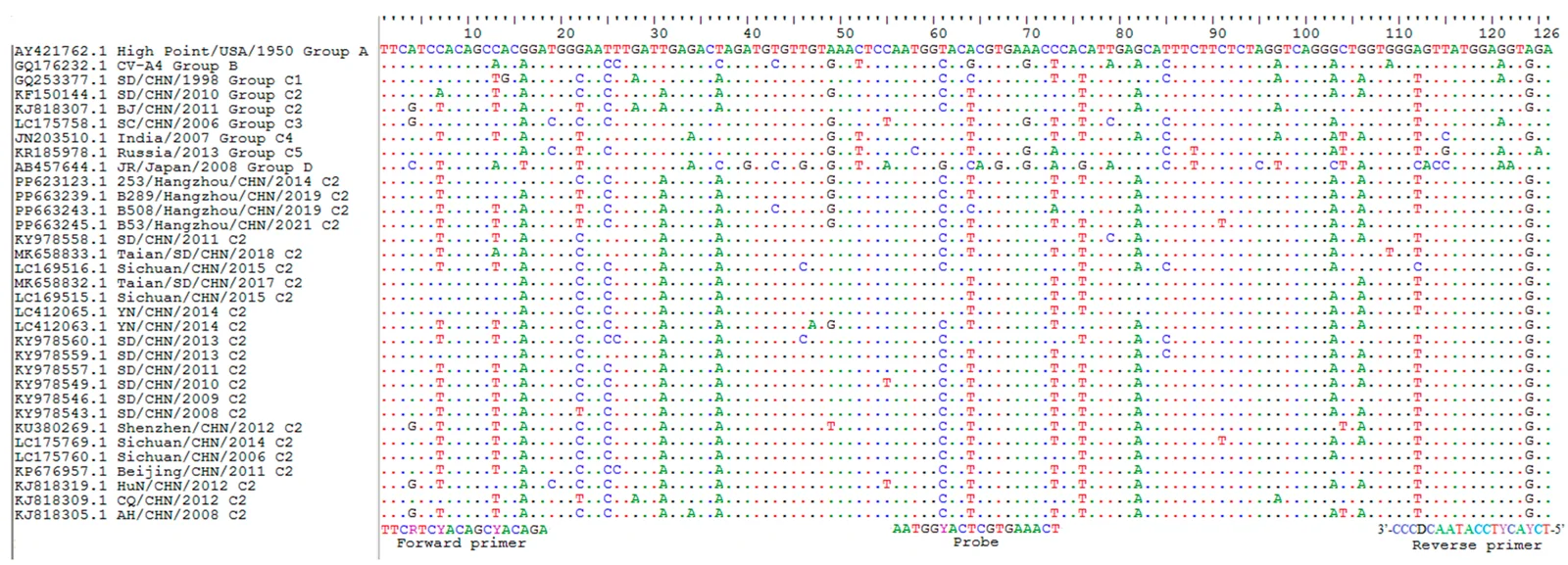
The Clustal W algorithm was used to align the VP1 genomic segments (126 bp) of several CV-A4 retrieved from GenBank. Primers and probe were displayed in the alignment. Notes: The VP1 gene fragments (126 bp) of 33 CV-A4 strains retrieved from GenBank were aligned using the Clustal W algorithm in MEGA 5.10 software. This region was highly conserved among representative strains of genotypes A–D and subgenotypes C1–C5, and the binding sites of both primers and the probe were located within the conserved region. The degenerate bases incorporated into the primers and probe (Y = T or C, R = G or A, D = G or A or T). The four nucleotide bases are color-coded for clarity: A (adenine) is shown in green, T (thymine) in red, C (cytosine) in blue, and G (guanine) in black. Dots indicate identity with the consensus sequence at the top of the alignment. The colors are used solely to distinguish the four bases and do not represent additional quantitative or functional information.

A total of 33 CV-A4 *VP1* nucleotide sequences were retrieved from GenBank and aligned using the Clustal W algorithm in MEGA 5.10 software with default parameters (Figure 1). The reference sequences encompassed genotypes A–D, with genotype C further divided into subgenotypes C1–C5, among which subgenotype C2 represents the predominant epidemic strain in China. Primers and TaqMan probes were designed within the relatively conserved *VP1* region using DNAStar and Primer Premier 5.0 software (Table 1). Primer and probe candidates were evaluated for optimal melting temperature, GC content, secondary structure, and dimer formation using Primer Premier 5.0. After screening 100 primer pairs, the best-performing set was selected (Supplementary Figure S1). Specificity was further verified by BLAST (version 2.15.0) analysis against the NCBI nucleotide database. The probe was 5′-labeled with 6-carboxyfluorescein (FAM) and 3′-quenched with Black Hole Quencher-1 (BHQ1), yielding an amplicon of 126 bp. All oligonucleotides were synthesized by Sangon Biotech Co., Ltd. (Shanghai, China).

### 2.5. RT-qPCR Assay

RT-qPCR was performed using the One-Step RT-PCR Kit (DRR064A, Takara Bio Inc., Kusatsu, Japan) on an ABI QuantStudio 5 Real-Time PCR System (Applied Biosystems, Life Technologies, Carlsbad, CA, USA). Each 20 µL reaction mixture comprised 10 µL of 2× RT-PCR buffer, 0.1–1 µL of each primer (1–10 nmol), 0.1–1 µL of probe (2–20 nmol), 0.4 µL of reverse transcriptase mix, 0.4 µL of polymerase, 5 µL of RNA template, and nuclease-free water to adjust the final volume. The stock concentrations of the primers and probe were 10 nmol/µL and 20 nmol/µL, respectively.

The primer reaction concentrations tested ranged from 0.05 to 0.5 nmol/µL, and probe reaction concentrations ranged from 0.1 to 1.0 nmol/µL. The optimal concentrations were selected based on cycle threshold (Ct) value and amplification efficiency. Standard curves for the optimization are provided in Supplementary Table S1. The optimal primer and probe reaction concentrations were 0.2 nmol/µL and 0.4 nmol/µL, respectively. The thermal cycling protocol was as follows: reverse transcription at 42 °C for 15 min; initial denaturation at 95 °C for 5 min; followed by 45 cycles of 95 °C for 5 s and 48–58 °C for 30 s. The optimal annealing temperature was determined to be 50 °C. Fluorescence signals were acquired in the FAM channel at the end of each extension step. Each run included a positive control (RNA extracted from CV-A4 strain 253/Hangzhou/CHN/2014 culture isolate) and a negative control (RNase-free water). Data were analyzed using QuantStudio 5 software, which generated standard curves by plotting Ct values against input CV-A4 concentration.

### 2.6. Establishment of Standard Curves

CV-A4 culture supernatant was thoroughly vortexed, and 200 µL (titer, 10^7.8^ TCID_50_/mL) was used for total nucleic acid extraction. The extracted nucleic acid was dissolved in 60 µL of elution buffer to obtain the CV-A4 nucleic acid stock. The stock was serially diluted 10-fold in RNase-free water to prepare calibration standards at dilutions of 10^−1^, 10^−2^, …, 10^−8^. Assuming 100% nucleic acid extraction recovery, the equivalent TCID_50_ concentration of the undiluted nucleic acid stock was calculated as follows: *C*_0_ = 200/60 × 10^7.8^ = 3.33 × 10^7.8^ = 2.1 × 10^8^ TCID_50_ equivalents/mL.

Standard dilutions showing a linear relationship (*R*^2^ > 0.99) between Ct values and template concentration, with consistent Ct values across replicates (*SD* < 1.0), were selected for standard curve generation. Standard curve generation was performed with three technical replicates per dilution and repeated in three independent experiments. In the QuantStudio 5 software, the detection channel was configured for standard samples, and the corresponding concentrations were entered. Each standard dilution was tested in triplicate, along with negative control wells. Standard curves were automatically generated upon completion of the run.

### 2.7. Specificity, Sensitivity, and Repeatability Tests

To evaluate specificity, nucleic acids extracted from clinical samples positive for CV-A2, CV-A4, CV-A5, CV-A6, CV-A8, CV-A10, CV-A16, EV-A71, CV-A9, CV-B1, CV-B3, E6, E7, E9, E11, E18, E21, E30, CV-A21, CV-A24, EV-D68, influenza virus, adenovirus, and rhinovirus were tested using the established RT-qPCR assay. Ct values and disease cases for all non-CV-A4 templates are provided in Supplementary Table S2. For experimental validation, available CV-A4 isolates of subgenotype C2 were used to verify the assay.

To assess sensitivity, 10-fold serial dilutions of the calibration standards (ranging from 0.21 to 2.1 × 10^8^ TCID_50_ equivalents/mL) were analyzed.

For repeatability evaluation, standard dilutions (2.1 to 2.1 × 10^8^ TCID_50_ equivalents/mL) were tested in triplicate, and the standard deviation (*SD*) and coefficient of variation (*CV*) of the Ct values were calculated. Data were analyzed using Microsoft Excel 2013 and IBM SPSS Statistics 21.0 (IBM, Corp., Armonk, NY, USA). Assay precision criteria were as follows: intra-assay *CV* < 5% and inter-assay *CV* < 10% were considered acceptable, based on the Minimum Information for Publication of Quantitative Real-Time PCR Experiments (MIQE) guidelines [35].

The limit of detection (LoD) of the RT-qPCR assay was determined in accordance with MIQE guidelines [35]. A 10-fold serial dilution series of the CV-A4 nucleic acid standard was prepared in RNase-free water, covering the range from the undiluted stock to the dilution at which amplification was no longer consistently observed. Each dilution was tested in a minimum of 10 replicates, with at least 4 no-template controls included per run. The LoD was defined as the lowest concentration at which ≥95% of the replicates yielded a positive amplification signal.

### 2.8. Preliminary Evaluation of the Developed Assay in Clinical Samples

From 2019 to 2023, a total of 82 CV-A4-positive and 100 CV-A4-negative clinical samples were collected from ILI patients in Hangzhou [17,33,34]. All positive samples were confirmed by conventional RT-PCR. The primers used for VP1 genotyping of CV-A4 were synthesized by Sangon Biotech Co., Ltd. (Shanghai, China), as previously described [36,37]. CV-A4-positive RNA samples were reverse-transcribed into cDNA using a reverse transcription kit (DRR6210A, Takara Bio, Shiga, Japan). The CV-A4 VP1 gene was amplified with a Takara ExTaq HS PCR kit (DRR006A, Takara Bio, Shiga, Japan), and the reaction conditions were set according to the kit instructions and previous references [36,37]. The PCR products were sequenced by Sangon Biotech (Shanghai). Sequence identity of the CV-A4 strains was determined using the BLAST search tool (http://www.ncbi.nlm.nih.gov/blast, accessed on 12 April 2026). The detection performance of the newly established method was compared with that of two commercially available CV-A4 RT-qPCR kits from different manufacturers. Commercial kits are based on the CV-A4 VP1 region and specific TaqMan probes for detecting amplicons.

## 3. Results

### 3.1. Establishment of an Optimal RT-qPCR Detection Method

Each 20 µL reaction mixture comprised 10 µL of 2× RT-PCR buffer, 0.4 µL of each primer (4 nmol), 0.4 µL of probe (8 nmol), 0.4 µL of reverse transcriptase mix, 0.4 µL of polymerase, 5 µL of RNA template, and nuclease-free water to adjust the final volume. The stock concentrations of the primers and probe were 10 nmol/µL and 20 nmol/µL, respectively.

The optimal thermal cycling protocol was as follows: reverse transcription at 42 °C for 15 min; initial denaturation at 95 °C for 5 min; followed by 45 cycles of 95 °C for 5 s and 50 °C for 30 s.

### 3.2. Standard Curve Establishment

**Figure 2:**
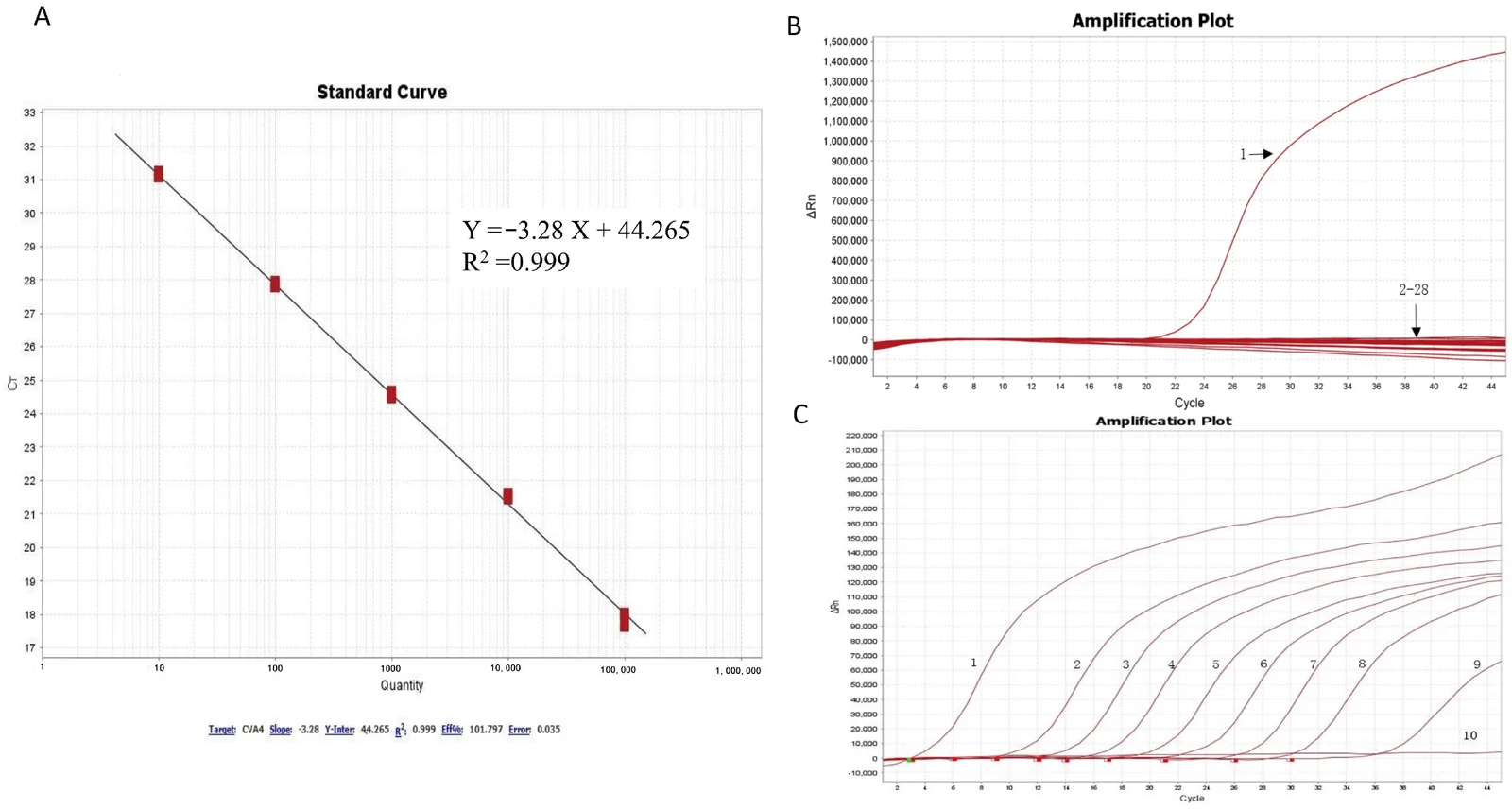
(**A**) Standard curves of the RT–qPCR. Standard concentrations were 21.00~2.10 × 10^5^ TCID_50_ equivalents/mL. (**B**) Specificity test of the RT–qPCR for detection of CV-A4. (**C**) The sensitivity test of the RT–qPCR for detection of CV-A4. Notes: Standard solutions with good linearity and repeatability were selected for curve generation. In the QuantStudio 5 software, the detection channel was configured for standard samples, and the corresponding concentrations were entered. Each standard dilution was tested in triplicate, along with negative control wells. Standard curves were automatically generated upon completion of the run. (**A**) Standard concentrations were 21.00~2.10 × 10^5^ TCID_50_ equivalents/mL. (**B**) 1: CV-A4 positive control; 2–28: CV-A2, CV-A4, CV-A5, CV-A6, CV-A8, CV-A10, CV-A16, EV-A71, CV-A9, CV-B1, CV-B3, E6, E7, E9, E11, E18, E21, E30, CV-A21, CV-A24, EV-D68, Influenza virus (H1N1, H3N2, BV, BY), Adenovirus, Rhinovirus-positive sample and negative control. (**C**) 1~9: Standard concentrations were 2.10~2.10 × 10^8^ TCID_50_ equivalents/mL, respectively; 10: 0.21 TCID_50_ equivalents/mL.

Serial 10-fold dilutions of CV-A4 nucleic acid extracts from viral isolates (nucleic acid, 21.0 to 2.1 × 10^5^ TCID_50_ equivalents/mL) were tested by RT-qPCR, and the resulting Ct values were used to generate a standard curve (Figure 2A). The standard curve equation was y = −3.28x + 44.265, with a correlation coefficient (*R*^2^) of 0.999, an amplification efficiency of 101.797%, and an error of 0.035.

### 3.3. Specificity, Sensitivity, and Reproducibility

As shown in Figure 2B, no amplification signals were observed for any of the non-CV-A4 viral nucleic acids tested, including other enteroviruses, adenovirus, and rhinovirus, confirming the high specificity of the assay.

For sensitivity, the assay reliably detected CV-A4 RNA across dilutions from 0.21 to 2.1 × 10^8^ TCID_50_ equivalents/mL, with a lower detection limit of 2.1 TCID_50_ equivalents/mL (Figure 2C). Following the MIQE guidelines, the LoD of the RT-qPCR assay was determined by endpoint dilution. The lowest concentration at which at least 95% of replicates tested positive was 2.1 TCID_50_ equivalents/mL. The corresponding Ct value was consistently below the threshold of 40. No amplification was observed in the no-template controls.

In reproducibility tests, serial dilutions from 2.1 to 2.1 × 10^8^ TCID_50_ equivalents/mL were tested in triplicate. Intra-assay *CV* < 5% and inter-assay *CV* < 10% were considered acceptable, based on MIQE guidelines [35]. Repeatability assessments showed low *SD* (≤0.44) and *CV* values (≤3.03%), confirming the stability and reliability of the assay (Table 2).

### 3.4. Detection of CV-A4 in Cell Culture and Clinical Samples

RNA extracted from CV-A4-infected cell cultures yielded positive results, while all negative controls remained negative.

In clinical testing, all 82 CV-A4-positive specimens collected from ILI patients in Hangzhou (2019–2023) were successfully detected by the established method, achieving a 100% detection rate within 1.5 h. Conventional RT-PCR and sequencing confirmed the positivity of all 82 specimens, with BLAST alignment against the NCBI database verifying the sequences as CV-A4. Of the 82 clinical samples, 22 representative samples covering different collection times and geographic locations were selected for sequencing and deposition in GenBank (accession numbers PP663239–PP663249 and PP738598–PP738608), as they were sufficient to confirm the assay specificity and the circulating C2 subgenotype.

### 3.5. Comparative Evaluation with Commercial Kits

When evaluated using 82 CV-A4-positive and 100 negative samples, the positive detection rates of the newly established method and the two CV-A4 commercial kits were 100% (82/82), 91.5% (75/82), and 89.0% (73/82), respectively. The corresponding false-positive rates were 0%, 0%, and 1% (Table 3). The single false-positive result obtained with one commercial kit was identified as CV-A10 by Conventional RT-PCR, sequencing and BLAST analysis. These findings indicate that the newly developed method exhibits superior sensitivity and specificity compared with the commercial kits evaluated and shows higher concordance with conventional RT-PCR results.

## 4. Discussion

CV-A4 is a common viral pathogen responsible for HFMD and herpangina in infants and can cause large-scale outbreaks within short time frames, posing a substantial threat to pediatric health [38,39]. Beyond these manifestations, CV-A4 has been implicated in severe conditions, including acute flaccid paralysis and viral meningitis [5,6].

The RT-qPCR assay established in this study exhibited excellent specificity, with no cross-reactivity observed against a comprehensive panel of enteroviruses (including *Enterovirus alphacoxsackie*, *Enterovirus betacoxsackie*, *Enterovirus coxsackiepol*, and *Enterovirus deconciliis* species), influenza viruses, adenoviruses, or rhinoviruses. The sensitivity reached 2.1 TCID_50_ equivalents/mL. The optimal annealing temperature was identified as 50 °C, as higher temperatures markedly reduced detection efficiency.

The predicted Tm of the TaqMan probe (59.1 °C) is close to that of the forward and reverse primers. We acknowledge that, according to standard TaqMan design guidelines, an ideal probe should have a Tm approximately 8–10 °C higher than the primers, preferably 68–70 °C. In the present assay, however, this was not an intentional design choice but a consequence of the highly conserved target region. Bioinformatic alignment of 33 target sequences showed that only an 18-nt window within the 126 bp *VP1* gene (CV-A4 prototype AY421762 position: 2607–2732) was 100% conserved among all target variants. Extending or shifting the probe by even a few bases introduced mismatches in the 33 target sequences and reduced the broad detection coverage required for this assay. In addition, because the probe contains a degenerate Y base, the Tm prediction software expands all possible sequences and reports an averaged Tm range rather than a single physical Tm; therefore, the predicted value may not fully reflect the effective Tm in the final reaction buffer.

To compensate for the suboptimal predicted probe Tm, we experimentally optimized the reaction conditions, including primer/probe concentrations, and annealing/extension temperature. Under the final conditions, the assay showed a standard curve slope of −3.28, *R*^2^ = 0.999, and an amplification efficiency of 101.797%, with a linear dynamic range of 21.0 to 2.1 × 10^5^ TCID_50_ equivalents/mL. The limit of detection was 2.1 TCID_50_ equivalents/mL, and no amplification was observed in no-template controls. The assay detected all 82 target strains and showed no cross-reactivity with 100 non-target pathogens. In clinical/simulated samples, the sensitivity and specificity were 100% and 100%, respectively, compared with the commercial kits.

Based on phylogenetic analysis of the *VP1* region, CV-A4 strains have been classified into four major genotypes (A–D), with genotype C further subdivided into C1–C5 [27]. In primer design, the reference sequences used in this study covered all major genotypes and subgenotypes, with particular emphasis on subgenotype C2, which represents the predominant circulating strain in China [3,4,17]. The coverage of the assay for genotypes A–D and subgenotypes C1–C5 was first clarified by in silico sequence analysis, which showed that the primer/probe binding sites are highly conserved across these lineages (Figure 1). For experimental validation, available isolates of subgenotype C2 were used. Notably, the newly developed assay demonstrated superior detection sensitivity and specificity for subgenotype C2 compared with commercial kits, underscoring its potential utility for CV-A4 surveillance and diagnosis in the Chinese context. Clinical isolates representing all genotypes/subgenotypes were not available for experimental testing.

Limitations: the assay was validated primarily with samples from China and may not perform equally well in other regions due to sequence variation; experimental validation was limited to the C2 subgenotype, whereas other genotypes/subgenotypes were only assessed in silico; only throat swab samples were evaluated, and other matrices (stool, serum, cerebrospinal fluid) were not tested; the number of clinical samples was moderate (*n* = 182), and larger multicenter studies are needed; mixed infections were not evaluated; and the assay’s performance may be affected by the emergence of new variants.

Since enterovirus types evolve rapidly through mutation and recombination, the diagnostic utility of this CV-A4-specific TaqMan assay is dependent on the clade currently circulating. The primer/probe binding sites were designed based on the predominant C2 subgenotype in China; however, the emergence of novel variants or recombination events within the *VP1* region could reduce assay sensitivity. Therefore, continued molecular surveillance and periodic re-evaluation of the assay are recommended. The combination of detection and typing in a single TaqMan assay provides a rapid and efficient tool for CV-A4 surveillance, but its broad applicability should be validated as the viral population evolves.

## 5. Conclusions

The TaqMan-based RT-qPCR assay established in this study provides a highly sensitive, specific, and reproducible method for the rapid detection of CV-A4, with superior performance compared with commercial kits, particularly for subgenotype C2. This method offers a promising tool for laboratory diagnosis and epidemiological surveillance of CV-A4 in China and other endemic regions.

## Figures and Tables

**Table 1:** Primers and probes used for the RT–qPCR.

Primer Name	Sequence(5′→3′)	Position *	Tm (°C)	Amplicon Size (bp)
Forward primer	5′-TTCRTCYACAGCYACAGA-3′	2607~2624	57.6	126
Reverse primer	5′-TCYACYTCCATAACDCCC-3′	2715~2732	55.7	
TaqMan probe	5′-FAM-AATGGYACTCGTGAAACT-BHQ1-3′	2662~2679	59.1	

* Notes: Y = T or C, R = G or A, D = G or A or T. Reference strain was CV-A4 prototype_High_Point/USA/1950. GenBank registration number: AY421762.

**Table 2:** Intra-assay and inter-assay reproducibility test of the RT–qPCR.

Concentration of CV-A4 (TCID_50_ Equivalents/mL)	n	Intra-Assay Variability (Ct)		Inter-Assay Variability (Ct)	
		$\bar{X}$ ± *SD*	*CV* (%)	$\bar{X}$	*CV* (%)
2.1 × 10^8^	3	4.273 ± 0.042	0.98	4.472 ± 0.068	1.52
2.1 × 10^7^	3	11.018 ± 0.183	1.66	11.725 ± 0.148	1.26
2.1 × 10^6^	3	14.530 ± 0.440	3.03	15.364 ± 0.194	1.26
2.1 × 10^5^	3	17.172 ± 0.130	0.76	17.472 ± 0.078	0.45
2.1 × 10^4^	3	20.300 ± 0.066	0.33	20.460 ± 0.114	0.48
2.1 × 10^3^	3	23.688 ± 0.209	0.88	23.687 ± 0.152	0.64
2.1 × 10^2^	3	27.421 ± 0.409	1.49	27.654 ± 0.084	0.30
2.1 × 10^1^	3	30.682 ± 0.247	0.81	30.850 ± 0.070	0.22
2.1 × 10^0^	3	37.143 ± 0.042	0.11	37.521 ± 0.053	0.14

**Table 3:** The result of CV-A4 detection in different assays.

Assays	Clinical Positive Samples	Detected Positive No. (Rate)	Clinical Negative Samples	Detected False-Positive No. (Rate)
This study	82	82 (100%)	100	0 (0.0%)
Commercial kit 1	82	75 (91.5%)	100	0 (0.0%)
Commercial kit 2	82	73 (89.0%)	100	1 (1.0%)

## Data Availability

No new data were generated or analyzed in support of this research. All data referenced are from previously published studies and are cited accordingly.

## Supplementary Materials

The following supporting information can be downloaded at: https://www.mdpi.com/article/10.3390/v18101120/s1, Figure S1: Primers and TaqMan probes were designed within the relatively conserved VP1 region (915bp) using Primer Premier 5.0 software; Table S1: Determination of optimal primer and probe concentrations for RT-qPCR; Table S2: Ct values for all non-CVA4 templates.
