# Supplementary material for: An RT–qPCR Assay for Rapid Detection of Coxsackievirus A4
Source: Viruses. 2026 Oct 9;18(10):1120. doi: 10.3390/v18101120 (PMC13709941; doi:10.3390/v18101120)
Supplement: Supplementary file 1 [file viruses-18-01120-s001.zip › viruses-4582219-supplementary.pdf]

Supplementary Materials

Table S1 Determination of optimal primer and probe concentrations for RT-qPCR

No.	Primer conc. (nmol/μL)	Probe conc. (nmol/μL)	CV-A4 RNA conc. 2.10×10^3 ($TCID_{50}$ equivalents/mL)		Standard curves	Efficiency (%)	*R²*	Error	NTC	LoD ($TCID_{50}$ equivalents /mL)
			Mean Ct ± *SD*	*CV* (%)						
**1**	0.5	1.0	27.62±0.50	1.81	y = −5.66x + 46.939	80.165	0.926	0.483	Negi ve	21
2	0.4	0.8	23.93±0.23	0.97	y = −3.90x + 39.525	90.238	0.942	0.306	Negi ve	21
3	0.2	0.4	23.69±0.21	0.88	y = −3.28x + 44.265	101.797	0.999	0.035	Negi ve	2.1
4	0.1	0.2	27.19±0.61	2.25	y = −4.45x + 44.785	87.877	0.981	0.198	Negi ve	21
5	0.05	0.1	34.10±0.55	1.60	y = −5.406x + 55.16	73.106	0.969	0.392	Negi ve	210

Note: Ct : Cycle threshold, *SD*: Standard Deviation, *CV*: Coefficient of variation, R^2: correlation coefficient, NTC: No Template Control, LoD: Limit of Detection.

Table S2 Ct values for all non-CVA4 templates

No.	Non-CVA4 templates	Ct values	Case Disease
1	CV-A2	25.4	Herpangina
2	CV-A4	26.5	Herpangina
3	CV-A5	26.8	Herpangina
4	CV-A6	28.3	HFMD
5	CV-A8	29.2	Herpangina
6	CV-A10	22.3	HFMD
7	CV-A16	29.1	HFMD
8	EV-A71	25.3	HFMD
9	CV-A9	30.7	Herpangina
10	CV-B1	31.8	ILI
11	CV-B3	28.7	ILI
12	E6	26.8	Encephalitis
13	E7	30.2	Encephalitis
14	E9	29.3	Encephalitis
15	E11	28.2	Bronchitis
16	E18	32.5	ILI
17	E21	30.2	ILI
18	E30	29.3	Encephalitis
19	CV-A21	33.0	ILI
20	CV-A24	28.4	Hemorrhagic conjunctivitis
21	EV-D68	30.2	ILI
22	H1N1	23.5	ILI
23	H3N2	28.9	ILI
24	BV	30.5	ILI
25	BY	32.7	ILI
26	Adenovirus	26.6	ILI
27	Rhinovirus	27.3	ILI

Note: HFMD: hand, foot, and mouth disease, ILI: influenza-like illness.

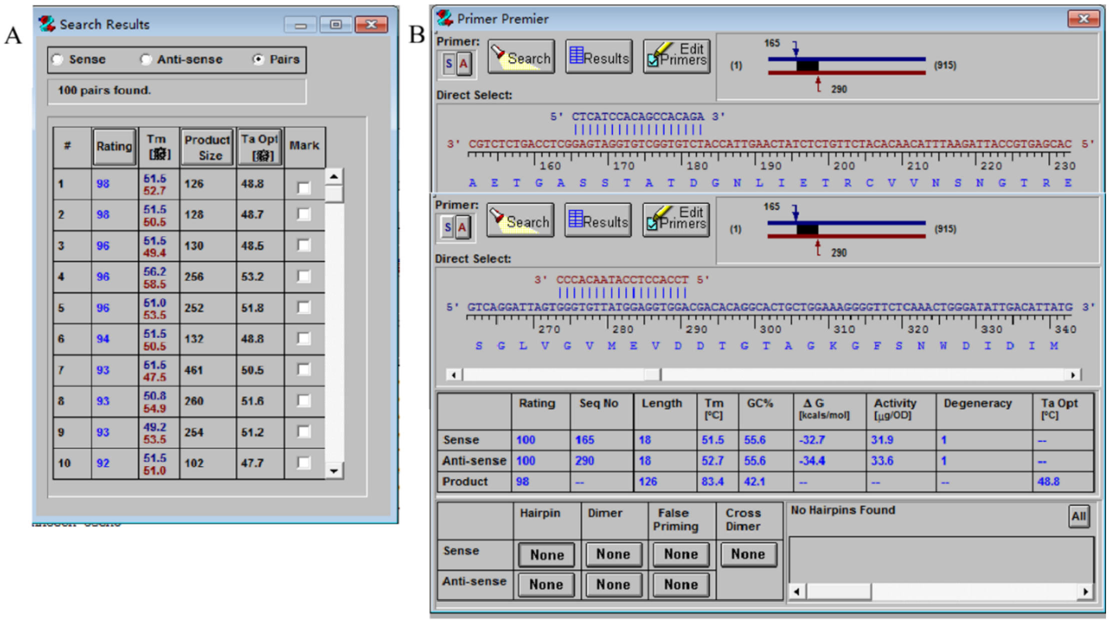

Figure S1 Primers and TaqMan probes were designed within the relatively conserved VP1 region（915bp）using Primer Premier 5.0 software. **Notes**: (**A**) The best-performing set was selected by screening 100 primer pairs. (**B**) Primer and probe candidates were evaluated for optimal melting temperature, GC content, secondary structure, and dimer formation using Primer Premier 5.0.
